# Caffeine: An Overview of Its Beneficial Effects in Experimental Models and Clinical Trials of Parkinson’s Disease

**DOI:** 10.3390/ijms21134766

**Published:** 2020-07-04

**Authors:** Giovanni Schepici, Serena Silvestro, Placido Bramanti, Emanuela Mazzon

**Affiliations:** IRCCS Centro Neurolesi “Bonino-Pulejo”, Via Provinciale Palermo, Contrada Casazza, 98124 Messina, Italy; giovanni.schepici@irccsme.it (G.S.); serena.silvestro@irccsme.it (S.S.); placido.bramanti@irccsme.it (P.B.)

**Keywords:** caffeine, Parkinson’s Disease, experimental models, neuroprotective effects, clinical trials

## Abstract

Parkinson’s Disease (PD) is a neurological disease characterized by the progressive degeneration of the nigrostriatal dopaminergic pathway with consequent loss of neurons in the substantia nigra pars compacta and dopamine depletion. The cytoplasmic inclusions of α-synuclein (α-Syn), known as Lewy bodies, are the cytologic hallmark of PD. The presence of α-Syn aggregates causes mitochondrial degeneration, responsible for the increase in oxidative stress and consequent neurodegeneration. PD is a progressive disease that shows a complicated pathogenesis. The current therapies are used to alleviate the symptoms of the disease without changing its clinical course. Recently, phytocompounds with neuroprotective effects and antioxidant properties such as caffeine have aroused the interest of researchers. The purpose of this review is to summarize the preclinical studies present in the literature and clinical trials recorded in ClinicalTrial.gov, aimed at illustrating the effects of caffeine used as a nutraceutical compound combined with the current PD therapies. Therefore, the preventive effects of caffeine in the neurodegeneration of dopaminergic neurons encourage the use of this alkaloid as a supplement to reduce the progress of the PD.

## 1. Introduction

PD is a neurodegenerative disorder characterized by the progressive neurodegeneration of the dopaminergic neurons in the substantia nigra pars compacta and in the corpus striatum [1]. The presence of α-Syn protein aggregates in neuronal cells, known as Lewy bodies, is a pathological hallmark of PD [2]. Clinically, PD is characterized by several symptoms such as tremors, postural instability, muscle stiffness, bradykinesia and akinesia. Moreover, the disease is supported by cognitive decline, anxiety, recurrent visual hallucinations, sleep disturbances, dementia and psychosis [3]. The etiopathogenesis of PD is multifactorial; indeed, genetic mutations and environmental factors are involved in the onset of the disease. The familial forms of PD are caused by autosomal dominant and recessive mutations in several genes such as α-Syn (*SNCA*), ubiquitin C-terminal hydrolase L1 (*UCHL-1*), phosphatase and tensin homolog-induced putative kinase 1 (*PINK1*), PARKIN (*PRKN*), protein deglycase (*DJ-1*) and leucine-rich repeat kinase 2 (*LRRK2*) [4]. Environmental factors such as exposure to herbicides; heavy metals; and pesticides including rotenone (ROT), dichlorodiphenyltrichloroethane, paraquat (PQ) and maneb (MB) are risk factors for the onset of idiopathic forms of PD [5].

To date, PD treatment involves the use of drugs capable of alleviating its symptoms [6]. Therefore, new therapies able to stop the progression of the disease are needed.

For these reasons, interest around natural compounds with neuroprotective properties such as caffeine has grown. Caffeine (1,3,7-trimethylxanthin) is a natural alkaloid extracted from coffee, tea and cocoa plants. This psychostimulant has several synonyms, such as theine, mateine and guaranine, although chemically it is the same molecule [7]. Caffeine is a compound capable of inhibiting lipid peroxidation as well as the formation of reactive oxygen species (ROS) [8]. Therefore, the constant consumption of caffeine acts on neurotoxicity by improving mitochondrial function and oxidative stress [9]. Although not fully clarified, the increase in oxidative stress in PD patients is supported by several factors that occur in the dopaminergic neurons. In particular, α-Syn aggregates could promote the formation of ROS, such as hydrogen peroxide (H_2_O_2_), superoxide anion and nitric oxide, and the consequent lipid peroxidation, DNA damage, increase in the reactive iron level and reduction in antioxidant species such as glutathione (GSH) and ascorbic acid. In this way, oxidative stress induces the apoptosis of neurons [10,11]. Moreover, mitochondrial dysfunction also plays an important role in the pathogenesis of PD [12]. Therefore, alterations in the mitochondrial Complex-I of the respiratory chain lead to a reduction in ATP synthesis and increase in ROS, thus favoring neurodegeneration [13].

In this regard, the neuroprotective action of caffeine has attracted considerable attention in the field of neurodegenerative diseases. Several studies have shown a relationship between regular caffeine consumption and a reduced risk of developing PD [14,15,16,17,18].

The aim of this manuscript was to provide an overview of experimental and clinical studies that report the neuroprotective role of caffeine in the PD. In order to write this review, a search was carried out in Pubmed using the following keywords: “caffeine”, “Parkinson’s Disease”, “experimental models”, “mice” and “rats”. We considered articles published between 2001 and 2020 demonstrating a neuroprotective role of caffeine in PD. We also provide a summary of current clinical trials recorded on ClinicalTrial.gov (https://clinicaltrials.gov/) using the following keywords: “caffeine”, “Parkinson’s Disease”.

## 2. Caffeine

**Figure 1:**
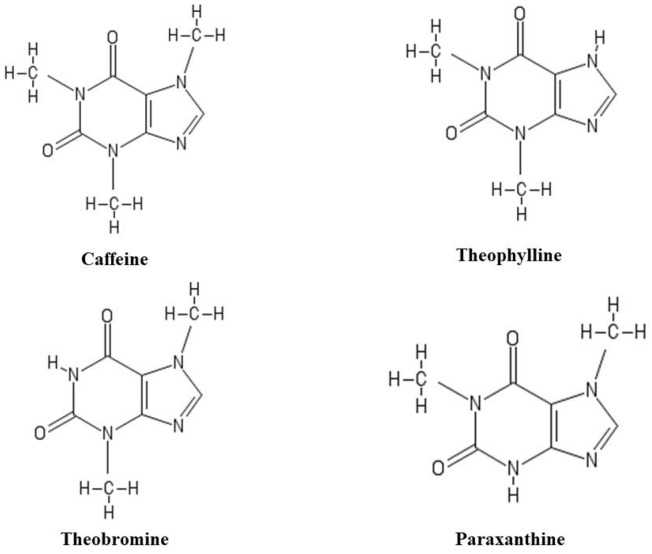
Chemical structure of caffeine and its three metabolites: theophylline, theobromine and paraxanthine.

Caffeine belongs to the methylxanthines family, a group of phytocompounds derived from xanthine. Caffeine is mainly extracted from the seeds and leaves of coffee (*Coffea arabica*), tea (*Camellia sinensis*) and cocoa (*Theobroma cacao L.*) [19]. Caffeine (1,3,7-trimethylxanthin) has a heterocyclic structure formed by pyrimidinedione and imidazole rings with imino nitrogen at position 9, which confers the property of weak acid. Additionally, it contains three methyl groups in positions 1, 3 and 7 [20]. Paraxanthin (1,7-dimethylxanthin) is the main metabolite of caffeine and also represents an isomer of theobromine (3,7-dimethylxanthin) and theophylline (1,3-dimethylxanthin). Unlike caffeine, paraxanthin lacks a methyl group at position 3 and theobromine lacks the methyl group at position 1, while theophylline is free of CH_3_ at position 7 (Figure 1). The presence of all three methyl groups gives the caffeine, physico-chemical properties different from other metabolites [21].

Tea and coffee are the most consumed drinks in the world and represent the main sources of caffeine taken through the diet [22]. Caffeine is absorbed in the gastrointestinal tract and metabolized in the liver by the cytochrome P450 enzyme system into three metabolites: paraxanthine, theobromine and theophylline [23]. Caffeine metabolites are present in different concentrations: 84% of paraxanthine, 12% of theobromine and 4% of theophylline [24,25]. Moreover, caffeine and paraxanthine exhibit a plasma half-life of 4.1 and 3.1 h; instead, theophylline and theobromine show a half-life of 6.2 and 7.2 h [26]. Caffeine and its metabolites cross all biological membranes and are distributed in all body fluids [27,28]. However, they do not accumulate in organs or tissues and are excreted in humans by urine [29]. Due to this liposolubility, caffeine crosses the blood-brain barrier, (BBB) influencing the central nervous system (CNS) and inducing respiratory and skeletal stimulation [21]. Conversely, the effects of paraxanthin in these systems can be considered irrelevant [30]. Paraxanthine plays an important role in the stimulation of lipolysis, leading to an increase in the concentration of fatty acids and glycerol in the blood, making them available for the muscles [31]. The mechanism used by paraxanthine involves the inhibition of the adenosine receptors (ARs) on the surface of the adipocytes [32]. Theobromine acts as a vasodilator prompting an increase in the concentration of nutrients and oxygen both in the muscles and brain. Additionally, it reduces blood pressure and stimulates diuresis. The mechanism of action of theobromine involves the inhibition of phosphodiesterase (PDE) as well as increasing the intracellular cyclic adenosine monophosphate (cAMP) with consequent vasodilator action [33]. Finally, theophylline, whose bronchodilator and diuretic properties can contribute to an increase in blood pressure and heart rate [34]. Theophylline competes with adenosine for Ars, whose inhibition stimulates the secretion of renin, thus promoting an increase in the glomerular filtration rate [35].

Generally, a moderate consumption of caffeine can have stimulatory effects on the CNS, improving wakefulness and attention span and reducing fatigue [36]. Moreover, a moderate consumption of caffeine can lead to a temporary increase in blood pressure and heart rate [37]. Instead, an excessive consumption of caffeine can induce adverse effects such as tachycardia, severe hypertension, nausea, vomiting, arrhythmia, convulsions and in some cases even death [38].

## 3. Neuroprotective Role of Caffeine in Experimental Studies of Parkinson’s Disease

**Figure 2:**
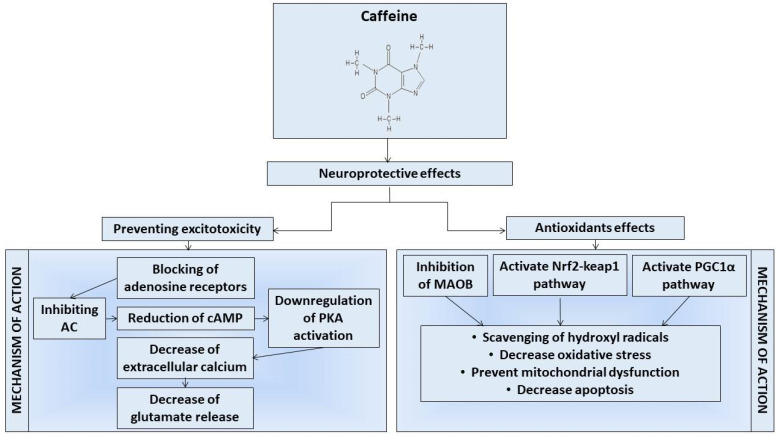
The image graphically summarizes the main mechanisms of action proposed to mediate the neuroprotective effects of caffeine.

Caffeine shows neuroprotective properties, and its beneficial effects can be related to antioxidant properties. Indeed, it has been observed that caffeine can inhibit lipid peroxidation by reducing the production of reactive oxygen species (ROS), such as hydroxyl radicals, H_2_O_2_ and singlet oxygen [39]. It can also exert its antioxidant effects by increasing the activity of glutathione S-transferase [40]. Specifically, in PD the high activity of monoamine oxidase-B (MAO-B) catalyzes the oxidation of dopamine by generating H_2_O_2_ [41]. In this way, the increase in oxidative stress contributes to the loss of dopaminergic neurons [39]. However, it has recently been shown that caffeine exerts its neuroprotective effects also through the inhibition of MAO-B (Figure 2). Therefore, the inhibition of MAO-Bs by caffeine could promote an increase in dopamine levels with the consequent improvement of motor symptoms. Moreover, it has been discovered that caffeine can protect dopaminergic neurons also due to the activation of the anti-oxidative signaling pathways, such as nuclear factor erythroid 2-related factor 2 (Nrf2)-Keap1 and peroxisome proliferator-activated receptor-gamma coactivator 1-α (PGC-1α), as illustrated in Figure 2 [42]. The Nrf2-Keap1 and PGC-1α pathways promote the expression of transcription factors involved in the biogenesis of mitochondria and in antioxidant and anti-inflammatory pathways [43,44]. Moreover, there was observed a loss of dopaminergic neurons in PGC1α knockout (KO) mice. On the contrary, in vitro the overexpression of PGC1α protects dopaminergic neurons from oxidative stress [45]. Therefore, by activating the signaling pathways Nrf2-keap1 and PGC-1α, caffeine could promote mitochondrial biogenesis, maintain redox homeostasis and promote cell proliferation.

Noteworthy, caffeine mediates its neuroprotective effects by modulating the action of the GABA receptor, regulating intracellular calcium, promoting the inhibition of PDE and antagonizing the ARs [46,47,48,49]. Several studies have shown that caffeine and theophylline modulate GABA receptors, acting as antagonists at benzodiazepine sites and interacting with GABA sites [50]. Caffeine is also involved in stimulating the release of intracellular calcium by agonizing the ryanodine receptors located in the sarcoplasmic reticulum [51]. However, compared to caffeine, theophylline, paraxanthin and theobromine appear to be less efficient in stimulating calcium channels [52]. Caffeine and its metabolites act as competitive PDE-4 inhibitors. The inhibition of PDE-4 promotes the hydrolysis of phosphodiester bonds in molecules such as cAMP, an important second messenger involved in cellular responses to neurotransmitters and hormones [53,54]. However, for caffeine to exert these mechanisms of action requires high concentrations that are difficult to reach with non-toxic consumption doses [55].

Nevertheless, the molecular structure of caffeine shows analogies with adenine and, indeed, caffeine acts as an antagonist for ARs: A_1_R, A_2A_R, A_2B_R and A_3_R [56]. ARs coupled with G proteins are present in different cells, such as immune cells, endothelial cells, blood vessels, microglia, astrocytes, corpus striatum and the spinal cord [57]. Normally, following the interaction between adenosine and A_1_R and A_3_R, the Gα_i_ protein is activated and in turn inhibits adenylate cyclase (AC). These events lead to a reduction in the production of cAMP with a consequent reduction in the activity of protein kinase A (PKA), which would otherwise phosphorylate calcium channels. Conversely, when adenosine binds to A_2A_R and A_2B_R, Gα_s_ protein is activated and induces an increase in AC, cAMP and PKA activity, thus promoting an increase in intracellular calcium [58,59]. Caffeine is an antagonist of all ARs. However, it has a higher affinity for A_1_R and A_2A_R [36].

Specifically, A_1_Rs are present in the cerebral cortex, in the cerebellum and in different parts of the brain [60]. Caffeine promotes the blockade of A_1_R [61]. However, A_1_R gene deletion or A_1_R blockade by caffeine does not induce changes in motor function. This proves that caffeine effects on motor activity are not mediated by its interaction with A_1_R [60]. Nevertheless, in rodents it was demonstrated that the chronic consumption of caffeine promotes the upregulation of adenosine A_1_R. The upregulation of A_1_R induces a decrease in pro-inflammatory cytokines, including the tumor necrosis factor-alpha (TNF-α). In this way, the interaction of caffeine with A_1_R could induce neuroprotective effects [62,63].

Conversely, A_2A_Rs are mainly located in the corpus striatum spiny neurons in the olfactory cortex, hippocampus and basal ganglia [61]. Therefore, the position of the A_2A_Rs favors a predisposition towards the adenosine–dopamine interaction responsible for most of the beneficial motor effects in PD induced by the A_2A_R antagonists. In order to confirm the important role of A_2A_R, it was shown how in the hippocampus of ischemic rats, the treatment with A_2A_R antagonists reduced the activation of the pERK1/2 pathway, as well as the levels of glutamate and consequently the inflammatory response [64]. Moreover, the inhibition of A_2A_R led to a reduction in TNF-α, nuclear factor-κB, prostaglandin E2 and interleukin-6 (IL-6), as well as an increase in anti-inflammatory cytokines such as interleukin-10 (IL-10) [65].

A_2A_R is also implicated as a modulator of inhibiting glutamate recovery, a neurotransmitter involved in the excitotoxicity of neurons [66,67,68]. Several studies have shown how, in pathological conditions, A_2A_R is responsible for the excessive release of glutamate [69,70,71]. The excessive release of glutamate can induce an increase in cytosolic calcium and inflammation events with consequent neuronal apoptosis [64]. In this regard, the modulatory action of A_2A_R antagonists such as caffeine in glial cells that involves the blocking or activating of these receptors can modulate the release of glutamate as well as neuronal death [72,73]. Therefore, caffeine, by blocking the binding site of adenosine to its receptor, negatively regulates the activity of PKA, leading to a reduction in intracellular calcium and the release of glutamate, also reducing the neuro-inflammatory processes and excitotoxicity (Figure 2) [74,75]. In line with these findings, several experimental studies have identified caffeine as a potential treatment capable of preventing or decreasing the neurodegeneration of dopaminergic neurons [76]. In order to support these findings, istradefylline, a selective A_2A_R antagonist, has been shown to improve the motor manifestations of PD [77]. Indeed, several randomized trials show that treatment with istradefylline for 20–80 mg/day for 6 weeks or up to 12 weeks has been well tolerated. Moreover, the treatment with istradefylline led to an improvement in the clinical symptoms of PD, especially tremors [78,79]. Additionally, combined treatment with istradefylline has prolonged the efficacy range of L-3,4-dihydroxyphenylalanine (L-DOPA) (76%; *p* < 0.05) [79]. Therefore, these results would confirm the involvement of A_2A_R in PD and highlight that drugs involved in block A_2A_R could be used to improve the symptoms of PD patients treated with L-DOPA.

Several studies suggest that caffeine binds to the α-Syn protein, inducing conformational changes and preventing aggregation, a hallmark of PD [80,81]. Generally, in PD the protein phosphatase 2A that dephosphorylates α-Syn is hypomethylated with a consequent reduction in the phosphatase activity. The reduction in the protein phosphatase 2A promotes the accumulation of hyperphosphorylated α-Syn. Yan et al. showed that caffeine protects the brain from α-Syn-mediated toxicity, maintaining the protein phosphatase 2A in an active state. In this study, the co-administration of caffeine and Eicosanoyl-5-hydroxytryptamide (for 6 months) in two different mouse models of α-synucleinopathies led to a reduction in phosphorylated α-Syn aggregates. This reduction preserved the neuronal function, decreasing the neuroinflammation with consequent behavioral improvements [82]. In line with these results, Luan et al. have evaluated the effect of caffeine in an α-Syn transmission model of PD induced by the intra-striatal injection of preformed α-Syn mutant fibrils (A53T). The PD model shows a loss of neurons and α-Syn inclusions similar to Lewy bodies and Lewy neurites. The authors observed that chronic caffeine consumption reduced α-Syn aggregates, neuroinflammation and cellular apoptosis in the corpus striatum [83]. Recent findings have shown that the blockade of A_2A_R reduces the aggregation of α-Syn in SynT-Synphilin-1 neuroglioma cells and improves synaptic and cognitive deficits in α-Syn-transgenic mice [84,85]. Moreover, the deletion of the A_2A_R gene prevents the loss of dopamine as well as dopaminergic neurons in a PD α-Syn model [86].

Consequently, the data obtained offer comforting evidence on the possibility that this alkaloid, due to its neuroprotective properties, in association with current drug therapies could offer benefits for PD patients [87].

## 4. Neuroprotective Role of Caffeine in Animal Models of Parkinson’s Disease

Several experimental studies have been performed to show the neuroprotective role of caffeine in the treatment of PD [88,89]. PD is caused by the loss of dopaminergic innervation in the corpus striatum, which can be experimentally induced by the use of neurotoxins such as N-methyl-4-phenyl-1,2,3,6-tetrahydropyridine (MPTP), 6-hydroxydopamine (6-OHDA) and ROT. These neurotoxins induce an increase in oxidative stress with the consequent progressive loss of dopaminergic neurons [90]. Moreover, MPTP is the main neurotoxin used in PD mice models able to cross the BBB [91]. Once in the brain, MPTP is converted into the active metabolite MPP+ within glial cells and serotonergic neurons. Therefore, MPP+ is released in the extracellular space and concentrated into dopaminergic neurons. In this way, MPP+ inhibits the mitochondrial Complex-I and increases ROS production, with a consequent reduction in dopamine levels in the corpus striatum [90,92]. Instead, the 6-OHDA model is induced with a single injection of this neurotoxin into the anterior brain of mice. The 6-OHDA shows a high mortality of dopaminergic neurons in the substantia nigra already after the first days of infusion [90,93]. ROT is a pesticide that inhibits mitochondrial Complex-I, inducing oxidative damage in dopaminergic neurons [90]. Additionally, it was shown that mice treated with low ROT concentrations for a prolonged period exhibit parkinsonian features such as motor deficit, the progressive loss of dopaminergic neurons and microglial activation [94]. These animal models of PD are useful for researchers to help discover new mechanisms underlying the disease as well as to find new neuroprotective treatments capable of preventing or stopping the progression of PD [95].

Chen et al. have evaluated the neuroprotective effect of caffeine in MPTP C57BL/6 mice. To better understand the role of caffeine, the authors also tested the effects of some A_2A_R-specific antagonists, including SCH 58261; 3,7-dimethyl-1-propargylxanthine (DMPX); istradefylline (KW-6002); and 8-cyclopentyl-1,3-dipropylxanthine, a specific antagonist of A_1_R. Caffeine attenuated the effect induced by MPTP in the dopaminergic nigrostriatal neurons. Moreover, caffeine at a dose of 20 mg/kg produced the maximum locomotor activity in MPTP C57BL/6 mice compared both to the control mice and mice lacking the A_2A_R. Additionally, even in mice lacking the A_2A_R, there was observed an improvement of MPTP-induced dopamine depletion. In conclusion, caffeine improved the survival of dopaminergic neurons in the MPTP model due to the blocking of A_2A_R, but not A_1_R blockers. Therefore, the mechanism whereby A_2A_R alters MPTP-induced neurotoxicity should be better clarified [96].

Sonsalla et al. have evaluated the neuroprotective effects of caffeine in a chronic progressive model of PD. MPTP induced a loss of nigrostriatal dopaminergic neurons in the corpus striatum. Treatment with caffeine after 7 and 21 days reduced the loss of nigrostriatal dopamine neurons. Therefore, this result showed that the administration of caffeine reduces neurodegeneration after the start of the damage. Moreover, striatal tyrosine hydroxylase staining revealed that caffeine consumption reduced the response of microglia in the substantia nigra but not in the corpus striatum. Thus, the study has shown that caffeine, in addition to blocking the A_2A_R, could exert its neuroprotective effects through the reduction in neuroinflammation in the substantia nigra, attenuating the microglial response and releasing pro-inflammatory cytokines in the lesion sites [75]. However, molecular studies are needed that would better explain how caffeine exerts these mechanisms.

The dysfunctions of the BBB play a key role in PD. In this regard, Chen et al. have evaluated the neuroprotective effect of caffeine by preventing damage to the BBB in male FVB mice. The authors showed that treatment with MPTP led to a reduction in tyrosine hydroxylase in dopaminergic neurons. Instead, caffeine has significantly reduced the loss of dopaminergic neurons. In order to evaluate the BBB selectivity, the level of occludin in tight junctions and microglial activity in the corpus striatum were quantified. Caffeine decreased the activation of astrocytes and microglia with a consequent reduction in the inflammatory processes, thus preventing the disruption of the BBB in the corpus striatum. The study showed that caffeine consumption stabilized the BBB due to a decrease in the neurodegeneration of dopaminergic neurons MPTP-induced through the reduction in neuroinflammation in the lesion sites. Nevertheless, the molecular mechanisms that describe how caffeine exerts its protective effects against harmful effects on the integrity of the BBB have not been explored in the study. Therefore, further studies are needed in order to clarify these mechanisms [97].

Singh et al. in MPTP mice compared the neuroprotective effects of caffeine and nicotine. Caffeine, compared to the control group, restored the transcription genes involved in several processes including cell apoptosis, cell cycle regulation and oxidative stress that appeared deregulated in MPTP mice. The same result was obtained in the nicotine group. Additionally, caffeine and nicotine restored the expression of glutamate receptor expression, calcium signaling and genes related to mitochondrial function. This study described the signaling pathways involved in MPTP-modulated neurodegeneration and those involved in caffeine and nicotine-mediated neuroprotection. However, further studies evaluating the involvement of these alkaloids in the individual molecular pathways are necessary [98].

The same researcher team, in another study, also investigated the effect of caffeine and nicotine in the modulation of the expression of genes associated with PD, including *VMAT-2*, *CYP1A1*, *CYP2E1*, *GST-ya*, *GST-yc* and *GSTA4-4*. The authors tested three different doses of both compounds. The results showed that only at the intermediate dose did caffeine (20 mg/kg) and nicotine (1 mg/kg) significantly prevent the depletion of dopamine levels induced by MPTP. Additionally, caffeine or nicotine at intermediate doses significantly reduced the expression of *CYP1A1*; the catalytic activity of Glutathione S-transferases; and the expression of the *GSTA4-4*, *GST-ya* and *GST-yc* genes increased by MPTP. Moreover, MPTP reduces the expression of *VMAT-2*, which, on the contrary, was increased by pretreatment with caffeine or nicotine. *VMAT-2* protects neurons from oxidative stress and damage induced by MPTP. Therefore, data obtained from biochemical investigations have shown that the interaction of caffeine and nicotine with these toxin-sensitive genes could be a strategy used by these compounds to prevent neuronal damage. However, molecular investigations would be needed to support these results [99].

Instead, Aguiar et al. have assessed the biochemical and behavioral changes induced by caffeine in 6-OHDA rats. Two weeks after treatment, the 6-OHDA induced motor deficits, the loss of dopaminergic neurons in the substantia nigra as well as a reduction in monoamines levels. Instead, caffeine consumption attenuated motor deficits and increased dopamine and monoamine levels. In line with these results, there has also been observed a significant reduction in the dopamine metabolite 3,4-dihydroxyphenylacetic acid (DOPAC), which was increased after the administration of caffeine. Therefore, these data suggesting the neuroprotective effects of caffeine could be mediated by its antagonistic action of A_2A_R; however, the mechanism has not been explored. Accordingly, new therapeutic strategies with more specific A_2A_R antagonists than caffeine may be useful for the treatment of PD [100].

In order to clarify this mechanism, the same researcher group tested the effects of 8-(-3-chlorostyryl)-caffeine (CSC), an antagonist of the A_2A_R and MAO-B inhibitor. In line with the previous study, the CSC treatment also attenuated motor dysfunctions and increased dopamine and DOPAC levels. Moreover, the effects of CSC were potentiated when administered together with L-DOPA. Similarly, CSC treatment promoted a decrease in monoamines, confirming its MOA-B inhibitor action. Furthermore, the authors also observed that the increase in glutamate and GABA in the corpus striatum, induced by 6-OHDA striatal lesion, was reduced after treatment with CSC. In the same way, the association with L-DOPA enhanced this effect. Additionally, the authors also showed that CSC reduced the levels of nitrite and lipid peroxidation in the corpus striatum, increased by the 6-OHDA striatal lesion. These data demonstrate that CSC performs its neuroprotective action both by restoring neurochemical alterations as an antagonist of A_2A_R and reducing oxidative stress. It has been also shown that the dual action of CSC as an A_2A_R antagonist and MAO-B inhibitor potentiates its neuroprotective effect. Although treatment with caffeine or other A_2A_R antagonists combined with L-DOPA has provided encouraging results, this mechanism should be better investigated [101].

In this context, Yu et al. have better tested the long-term interaction between caffeine and L-DOPA in 6-OHDA mice. The results have shown that only treatment with L-DOPA or caffeine provided a different behavioral response. Conversely, daily treatment with caffeine did not show an improvement in sensitized rotational behavior. However, two weeks after stopping the treatment, the mice underwent a new treatment with L-DOPA or caffeine. The authors showed that repeated treatment with L-DOPA induced the loss of sensitized rotation. On the contrary, repeated treatment with caffeine showed an improvement in sensitized rotational behavior. These differential results suggest that long-term adaptive mechanisms could be activated. To better understand the cross-effect between caffeine and L-DOPA, the authors co-administered caffeine (10 mg/kg) and L-dopa (2 mg/kg) in 6-OHDA mice for 26 days. The synergistic administration of L-DOPA and caffeine has improved the sensitized rotational behavior compared to single uses of L-DOPA or caffeine. Therefore, the pharmacological cross-actions and the synergistic interactions between caffeine and L-DOPA show that caffeine could change the responses of L-DOPA induced in PD [102].

Reyhani-Rad et al. aimed to evaluate the effect of caffeine on 6-OHDA-induced motor disorder by antagonizing A_2A_R. In order to investigate these mechanisms, mice were treated with caffeine or SCH 58261, an A_2A_R antagonist. The authors observed that, after three weeks, the treatment with 6-OHDA led to balance disorders. Nevertheless, the treatments with caffeine at a dose of 30 mg/kg or SCH 5826 10, 30 and 60 min after administration induced a significant improvement in balance disorders. Moreover, high doses of caffeine or SCH 58261 can lead to improved motor deficits and bradykinesia induced by 6-OHDA. In conclusion, the study has shown that the neuroprotective effect of caffeine, as well as A_2A_R antagonists, could be explained through the inhibition of presynaptic A_2A_R [103].

To understand how caffeine and A_2A_R and A_1_R-selective antagonists can improve damage induced in PD, Kelsey et al. have assessed the effects of caffeine and selective A_2A_R and A_1_R antagonists on motor deficits induced by 6-OHDA. For this purpose, after the 6-OHDA-induced injury rats were subjected to the systemic administration of caffeine and L-DOPA, SCH 58261 and L-DOPA, N^6^-Cyclopentyladenosine (A_1_R antagonist) and 8-Cyclopentyltheophylline (A_1_R antagonist). Twenty minutes after the administration of the drugs, the mice were subjected to the stepping test. The 6-OHDA induced a significant reduction in the frequency of stepping, which increased after the administration of caffeine and SCH 58261. Additionally, these A_2A_R antagonists also enhanced the effect of L-DOPA. Contrarily, the A_1_R antagonists did not have any effect on motor functions. Moreover, the effectiveness of caffeine (20–40 micrograms) directly administered into the dorsal striatum or in the external globus pallidus was also evaluated. The administration of caffeine in these sites also improved the motor deficits induced by 6-OHDA. Therefore, these data confirm the effectiveness of A_2A_R antagonists in the common treatment of PD. Additionally, the study even suggests that the dorsal or globus pallidus striatum may be potential sites for caffeine administration [104].

To date, treatment with L-DOPA is the treatment most used for PD; however, prolonged treatment could induce dyskinesia in PD patients. In this regard, Jones et al. aimed to evaluate whether chronic treatment with A_2A_R antagonists induced the same effects. In order to evaluate this aspect, 14 days after the 6-OHDA-induced lesion rats were treated with A_2A_R antagonists, including SCH 412348, vipadenant, caffeine and istradefillin. The results showed that, after 21 days of treatment, none of the A_2A_R antagonists induced dyskinesias in 6-OHDA rats. In order to further verify these data, the authors sensitized the 6-OHDA rats with L-DOPA at a dose of 6 mg/kg daily until a dyskinesia profile was reached. The day after receiving the final dose of L-DOPA, the rats were treated with a single dose of SCH 412348. The co-administration of the A_2A_R antagonist with L-DOPA did not reduce the L-DOPA-induced dyskinesia. These results suggesting that A_2A_R antagonists cannot induce dyskinesia in humans, but contrarily they can show a beneficial anti-dyskinetic potential. However, if co-administered with L-DOPA they may not block the effects induced by L-DOPA. Thereby, it is necessary to better clarify in clinical studies the role of A_2A_R antagonists on dyskinesia behaviors [105].

In order to understand the role of ARs in L-DOPA-induced dyskinesia, Xiao et al. have aimed to evaluate the effects of the gene deletion of these receptors. Therefore, in the study KO mice were used for both A_1_R and A_2A_R and double A_1_R-A_2A_R KO. The study results showed that following 6-OHDA-induced injury, receptor blockade induced by genetic deletion or caffeine did not change the dopamine or DOPAC levels in the corpus striatum. Behavioral tests involving the recording of contralateral rotations and dyskinesia by quantifying abnormal involuntary movements (AIMs) showed that, on days 11–21, the treatment with L-DOPA induced an increase in contralateral rotations and AIMs. Conversely, the genetic blockade of the receptors showed an attenuation of the contralateral rotations in all three genotypes of mice. However, the attenuation of AIMs was observed in A_1_R KO and A_2A_R KO mice. In contrast, the A_1_R-A_2A_R double KO mice showed high levels of AIMs. Instead, the pharmacological blockade of the receptors with caffeine did not induce the attenuation of the contralateral rotations or dyskinesia in either of the two doses. Therefore, the study shows that the blocking of A_1_R or A_2A_R could be used as a possible therapeutic strategy to improve the effects of dyskinesia induced by L-DOPA in PD patients. However, the effect of the combined inhibition of both receptors remains to be clarified [106].

Khadrawy et al. showed in ROT rats the effects of caffeine on histopathological, behavioral and neurochemical alterations. ROT has led to a decline in motor activity and muscle strength. Additionally, ROT promoted oxidative stress, which was measured by the increase in malondialdehyde (MDA) and nitric oxide in the midbrain and corpus striatum of rats. Moreover, it reduced the levels of GSH, SOD, GSH-S-transferase, acetylcholinesterase and Na^+^/K^+^-ATPase. Consequently, an increase in TNF-α in the midbrain and corpus striatum of these animals was shown. Conversely, the administration of caffeine led to a significant reduction in MDA as well as a decrease in oxidative stress, and improved the changes in acetylcholinesterase and Na^+^/K^+^-ATPase activities. Additionally, caffeine restored dopamine levels, thus preventing the motor deficits induced by ROT. In conclusion, this study highlighted the antioxidant power of caffeine in the corpus striatum and midbrain following the increase in ROT-induced lipid peroxidation [107].

PQ and MB are herbicides often used together that are linked as potential risk factors in PD. In this regard, Kachroo et al. induced a model of environmental parkinsonism in C57BL/6NCrl mice using a double pesticide to evaluate the effects of caffeine. Motor evaluations performed 24–48 h after each treatment showed that none of the treatments induced differences in motor activity. Subsequently, a week after the end of the treatment, the stereological evaluation of the neuronal loss in the substantia nigra pars compacta, evaluated by the immunoreactivity of tyrosine hydroxylase, showed that exposure to the two herbicides reduced the number of neurons. In contrast, caffeine at a dose of 20 mg/kg has significantly reduced neuronal loss. The results showed that the long-term pretreatment of caffeine appears to protect dopaminergic neurons. Therefore, the neuroprotective role of caffeine, even in a chronic PD model, reinforces the hypothesis that this alkaloid could be a valid therapeutic tool to slow down the degenerative process underlying PD [108].

A further mice model of PD is induced with reserpine at doses of 1–5 mg/kg. Reserpine is an alkaloid with antihypertensive and antipsychotic properties; it interferes at the vesicular level with the conversion of monoamines, causing depletion in the nerve terminals and consequent muscle rigidity. In catecholaminergic neurons, reserpine is an irreversible inhibitor of the vesicular monoamine transporters, whose blockage leads to the exhaustion of catecholamines from the vesicles. Moreover, non-vesicular catecholamines are rapidly metabolized by MAO, and hence reserpine reduces the levels of neurotransmitters such as norepinephrine, serotonin and dopamine and their subsequent release [95,109,110]. Moo-Puc et al. have evaluated the effects of caffeine on memory deficits caused by treatment with high doses of trihexyphenidyl (THP) in reserpine rat models. A single treatment with caffeine or THP did not invert hypokinesia induced by reserpine. Conversely, in reserpine rats, the combined treatment of caffeine and THP led to levels of motor activity like the one shown in the control group. Moreover, it was demonstrated that, in reserpine rats, both treatment with low doses of caffeine or THP led to the full recovery of motor and exploratory activities, which were likely reduced due to dopamine depletion induced by reserpine. Therefore, it was demonstrated how the combined treatment of caffeine and THP is potentially useful in alleviating motor deficits, thus avoiding the memory deficits induced by treatment with high doses of THP [111].

In Table 1 are listed studies of animal models that show the neuroprotective effects of caffeine in the treatment of PD.

## 5. Evaluation of the Effects of Caffeine in Clinical Trials of Parkinson’s Disease

In recent decades, several studies have been performed to evaluate the effects of caffeine as a nutraceuticals compound in PD. This section offers a summary of the clinical trials registered on https://clinicaltrials.gov/ Moreover, it provides a summary of the recent prospective studies recorded in Pubmed.

A completed Phase 2 clinical study (NCT01190735) was performed on 28 patients (aged 18 years and older) with idiopathic PD diagnosed by the Hoehn and Yahr scale (stage I–IV). The aim of the study was to evaluate both the tolerability and efficacy of the treatment at increasing doses of caffeine. Each patient received twice a day treatment for six consecutive weeks with prepackaged caffeine pills at increasing doses: 100 mg during the first week, 200 mg in the second week, 300 mg in the third week and 500 mg during the last two weeks. During the study, the patients continued drug therapy for PD. The results of these studies are not yet available; however, they will be useful to establish the optimal caffeine dose responsible for the motor benefits and the least amount of adverse events.

The same researcher team, in a double-blind, controlled phase 2/3 complete study (NCT00459420), also evaluated the effects of caffeine in 58 patients (aged 18 years and older) with idiopathic PD. The participants were randomized and treated for the first 3 weeks twice a day with caffeine at a dose of 100 mg and for the following 3 weeks with caffeine at a dose of 200 mg. Meanwhile, another group took a placebo twice a day (control group). After 6 weeks, to prevent withdrawal symptoms the patients were treated with caffeine (100 mg) one week. Additionally, the participants continued their PD drug therapy and were also accustomed to consuming caffeinated drinks. Treatment with caffeine after three weeks did not lead to a significant improvement in the Epworth Sleepiness Scale (ESS) score compared to the placebo group. After 6 weeks, there was observed a reduction in the ESS score. Therefore, the consumption of caffeine did not reduce sleep loss, nor a deterioration in the quality of the night’s sleep, and did not affect mood. However, caffeine was well-tolerated without side effects. Additionally, the caffeine consumption after 6 weeks showed a significant improvement in the Clinical Global Impression of Change outcomes compared to the placebo group. Moreover, the patients who received caffeine supplements demonstrated a five-point improvement in the Unified Parkinson’s Disease Rating Scale (UPDRS), an indicator commonly used to assess disease severity. In conclusion, there was not observed a benefit of caffeine upon excessive daytime somnolence in PD patients, despite there being identified an improvement in motor manifestations. However, the duration of the study was short, therefore the results of the study need long-term evaluation [112].

These potential motor benefits were better explored in a larger long-term trial (NCT01738178). In this trial, 121 patients (aged 45–70 years) who were affected by idiopathic PD diagnosed as parkinsonism according to the UK brain bank criteria were enrolled. The inclusion criteria in the study were disease duration from 6 months to 8 years, Hoehn and Yahr stage I–III and the use of stable therapy for PD for at least 6 months. The patients received 200 mg caffeine capsules (or placebo) twice a day in the morning and after lunch (the dose corresponds to about 3 cups of coffee per day). In order to improve the tolerability and reduce the adverse effects, the caffeine dose was increased by 50 mg per week until the full dose was reached at week 9. After 6 months, the patients continued to employ caffeine or placebo for a further 12 months. Caffeine administration did not cause adverse events compared to the control group. However, improvements in motor parkinsonism were not observed during the study in patients who took caffeine compared to patients in the control group. Conversely, caffeine intake led to a slight improvement in sleepiness in the first 6 months, which eased during the study. Instead, both after one year and after 18 months, the patients taking caffeine had a slight increase in dyskinesia. However, the prolonged administration of caffeine caused a worsening of cognitive tests. In conclusion, the results of the study showed that caffeine did not provide motor improvements in PD patients. Therefore, future studies are needed to elucidate the epidemiological links between caffeine intake and low PD risk [113].

In order to report the beneficial effects of caffeine in PD, several prospective studies were performed. Ascherio et al. in a prospective cohort study evaluated the relationship between coffee and caffeine consumption with the risk of PD. The study included two cohorts of participants: the Follow-up Study of Health Professionals (HPFS) and the Nurses’ Health Study (NHS). The authors collected data on eating habits and lifestyle during a follow-up of 10 years for men and 16 for women through a questionnaire. The study results showed that men with moderate coffee consumption have a lower risk of PD disease than men who never drink coffee. The same result was also obtained in the participants who consumed caffeine derived from sources other than coffee. Instead, the opposite result was observed in the participants who consumed decaffeinated coffee. This evidence highlights that the inverse relationship between caffeine consumption and PD risk is due to caffeine and not to other coffee compounds. Conversely, in women the lowest risk of PD was observed following a low consumption of coffee (1–3 cups of coffee per day) [17]. In a subsequent prospective study, Ascherio et al. evaluated in 909 men and 340 women the relationship between caffeine consumption and mortality rate for PD. In the results obtained, an inverse relationship between mortality and caffeine consumption was observed, confirming as in the previous study the neuroprotective role of this alkaloid in PD. Additionally, it was shown that cigarette smoking and alcohol consumption along with caffeine has led to a reduced risk of PD. However, this inverse relationship was not observed in women who used estrogen. Contrarily, caffeine consumption was associated with reduced PD mortality in women who had never used postmenopausal estrogen. Since the main limitations of the study were the absence of information on the diagnosis of PD among the participants and on the changes in coffee consumption or in the use of estrogen during follow-up, further studies are needed to evaluate the effects of caffeine or estrogen on the progression of PD [114].

For this purpose, Palacios et al. have evaluated the effects of caffeine consumption in 197 men and 120 women. The results of the study showed that the risk of PD decreased significantly with the increase in caffeine consumption. Moreover, it was demonstrated that the PD risk was lower in men and higher in women. In men, regular caffeine intake (two or more cups per day) was associated with a lower risk of PD compared to non-coffee consumers. A similar result has also been achieved in women. However, in women, the reduced inverse relationship between caffeine consumption and PD risk could probably be due to an interaction with hormone therapy. Indeed, the study data reported a higher risk of PD in women undergoing hormone therapy compared to non-users of therapy. This result confirms that hormone therapy influences the beneficial effects of taking caffeine. Although this study includes prospective data with 8 years of follow-up, the low caffeine intake in this cohort did not allow the researchers to study the effects caused by high doses of coffee [115].

In this regard, Bakshi et al. assessed the effects of higher coffee consumption in 369 patients with idiopathic PD and 197 healthy controls (HC) included among participants in the Harvard Biomarkers Study. Caffeine consumption was evaluated via a semi-quantitative questionnaire during the previous 12 months, with a possible consumption of coffee which varied from never to 6 or more cups per day. The results of the study showed that caffeine consumption was lower in the PD patients compared to the HC. Moreover, it was reported that a high caffeine level was associated with a lower risk of idiopathic PD in a sex-independent way. Therefore, habitual caffeine consumption can have beneficial effects on humans by decreasing the risk of PD [116].

The correlation between habitual caffeine consumption and decreased risk of PD was also assessed in a potential cohort of 8004 Japanese-American men with 30 years of follow-up. In this study, Ross et al. reported a lower risk of PD proportional to the increase in caffeine consumed. Moreover, non-coffee consumers showed a PD risk five times greater compared to patients who consumed 28 ounces or more daily. In this study, was also observed that the risk of PD was 2 to 3 times greater in smokers who did not consume coffee compared to coffee drinkers. Therefore, this prospective long-follow-up study has shown that coffee consumption is inversely related to the risk of PD, regardless of smoking [15].

Coffee consumption also reduced the risk of PD in a case-control study of 395 PD patients conducted by Paganini-Hill et al. Contrary to the previous study, in this study smoking also reduced the risk of PD. Moreover, other risk factors such as alcohol consumption, hypertension, the number of children and the total intake of vitamin A and vitamin C were also assessed. Based on the data obtained, a reduction in the risk of PD among alcohol consumers was also observed. Contrarily, a significant increase in disease risk has been demonstrated in patients with more than three children and a diet with an extra intake of vitamins A and C. These results highlight the direct link between lifestyle and the development of PD [117].

Other cohort studies have evaluated the possibility that caffeine may be protective in PD. Specifically, Hu et al. evaluated this relationship in a cohort of 200 PD patients for an average period of 12.9 years. The results of the study showed a lower risk in PD patients who habitually consumed caffeine at the dose of (three or more cups of tea) or coffee (1–4 cups or more) daily. Moreover, it was reported that higher caffeine consumption reduced the risk of PD, mostly in the 25–49 age range rather than in the 50–74 age range [118].

Instead, Sääksjärvi et al. have assessed the relationship between coffee consumption and PD for 22 years in 101 PD patients. The results of the study reported a lower risk of PD in daily consumers of 10 cups of coffee compared to non-coffee drinkers [16].

The same result was also observed from Tan et al. in 157 PD patients of both sexes. Additionally, in this study the reduced risk of PD was also associated with the consumption of black tea but not green tea, regardless of cigarette smoking and total caffeine consumption. The protective effect of black tea could be attributed to its caffeine content [119]. In line with these results, the inverse association between caffeine and PD risk was also highlighted in a meta-analysis study conducted mainly in western populations. In this study, Hernán et al. in eight case-control studies and four cohort studies observed that daily coffee consumers, compared to non-consumers, had a lower risk of PD [120]. In this regard, Kyrozis et al. showed the role of caffeine, eating habits and lifestyle in 118 Greek PD patients. The authors reported that caffeine consumption was related to a lower risk of PD. Moreover, it was observed that the intake of other foods such as milk but not its derivatives and the intake of polyunsaturated fats seem to confer more PD risk [121]. Instead, Liu et al. in a cohort of 318,260 participants observed the inverse relationship between the intake of caffeine and a lower risk of PD. However, there were no shown differences in gender in the association between caffeine and PD risk [122].

In Table 2 are listed clinical studies that show the neuroprotective effects of caffeine in PD patients and prospective studies that highlight the inverse correlation between the consumption of caffeine and PD risk.

## 6. Conclusions

The purpose of this review was to provide an overview of the experimental and clinical studies that described the protective effects of caffeine consumption in the PD. The most common PD animal models involve the use of neurotoxins such as MPTP and 6-OHDA, which induce the selective destruction of the dopaminergic pathways by reproducing the specific characteristics of PD in rodents. The results of the studies suggested that the adenosine receptor antagonism, in particular A_1_R and/or A_2A_R, is the mechanism mainly responsible for the neuroprotective effects of caffeine. However, the protection against BBB alteration could be considered an additional mechanism of action that justifies these beneficial effects. Therefore, these findings support the development of A_2A_R antagonist antiparkinsonian drugs.

In compliance with these results, clinical and prospective studies have shown the preventive effects of caffeine in the neurodegeneration of dopaminergic neurons. However, longitudinal studies involving the recruitment of a larger cohort of patients are needed to confirm these findings and to better explore the use of caffeine as a supplement to reduce the progress of PD.

## Figures and Tables

**Table 1:** Summary of the preclinical studies that evaluate the neuroprotective effects of caffeine in the treatment of PD. Specifically, the table lists the animal models used in the studies, the type of treatment, the dosage and the therapeutic effects obtained.

Animal Models	Induced PD Models	Treatment	Dosage and Route of Administration	Therapeutic Effects	Ref.
C57BL/6 mice	MPTP (single dose 40 mg/kg) or (multiple-dose 20 mg/kg) via intraperitoneal	Caffeine and A_2A_R antagonists	0–60 mg/kg via intraperitoneal 10 min before injury	Caffeine reduced MPTP-induced dopamine depletion, improving the locomotor activity. Caffeine exerts its action blocking the A_2A_Rs, as demonstrated by using mice lacking the A_2A_R. The treatment with A_2A_R antagonists also confirmed this result.	[96]
male Sprague–Dawley rats	MPTP (75 μg/ day) unilateral intra-cerebroventricular infusion for 28 days	Caffeine	60–80 mg/kg daily, after 1 to 3 weeks from the injury	After 7 and 21 days of caffeine administration, mice treated with MPTP showed a reduction in the loss of nigrostriatal dopamine neurons and the response of microglia in the substantia nigra, with a consequent decrease in neuroinflammation.	[75]
FVB mice	MPTP (20 mg/kg) daily via intraperitoneal for the second week	Caffeine	10 mg/kg via intraperitoneal for the first 7 days and 10 min before MPTP treatment for the second week	The pretreatment with caffeine prevented the damage to the BBB induced by MPTP and decreased the activation of astrocytes and microglia.	[97]
Swiss albino mice	MPTP (20 mg/kg) daily for the subsequently 4 weeks	Caffeine and nicotine	Caffeine (20 mg/kg) and nicotine (1 mg/kg), via intraperitoneal, daily for the first 8 weeks	The consumption of caffeine restored the transcription of genes involved in several processes, including cell apoptosis, cell cycle regulation and oxidative stress. These transcripts were downregulated in MPTP mice.	[98]
Swiss albino mice	MPTP (20 mg/kg) from 1 day to 4 weeks	Caffeine and nicotine	Caffeine (10–30 mg/kg) and nicotine (0.5–1.5 mg/kg) pretreated daily via intraperitoneal for 8 weeks	Caffeine (20 mg/kg) or nicotine (1 mg/kg) treatments prevented the depletion of dopamine induced by MPTP. Both alkaloids reduced the expression of *GSTA4-4*, *GST-ya*, *GST-yc* and *VMAT-2*, reducing the MPTP-induced toxicity.	[99]
Wistar rats	6-OHDA (12 μg/μL) single stereotaxic injection	Caffeine	10 and 20 mg/kg via intraperitoneal for 13 days, one hour after injury	Caffeine prevented the MPTP-induced dopamine and DOPAC depletion. In this way, caffeine improved motor deficits.	[100]
Wistar rats	6-OHDA (12 μg/μL) stereotaxic injection into the right corpus striatum	CSC and L-DOPA	Caffeine 1–5 mg/kg and L-DOPA (50 mg/kg + benserazide 12.5 mg/kg) administered alone or co-administered for 7 consecutive days	The CSC prevented dopamine depletion and DOPAC, 6-OHDA-induced. The CSC treatment promoted a decrease in monoamines, enhancing its neuroprotective effect. The effects of CSC were potentiated when administered together with L-DOPA.	[101]
C57BL/6 mice	6-OHDA (2.5 μg/μL) unilaterally into the left dorsal corpus striatum	Caffeine and L-DOPA	Caffeine (2.5 or 10 mg/kg) and L-DOPA (2.0 mg /kg) administered alone via intraperitoneal after the injury for 14–21 days, or co-administered at higher dosage for 26 days	The treatment of caffeine associated with L-DOPA led to an improvement in sensitized rotational behavior in mice induced with 6-OHDA compared to control mice.	[102]
Wistar rats	6-OHDA (0.2 μL/min) unilaterally into the substantia nigra	Caffeine and SCH 5826	Caffeine (30 mg/kg) and SCH 58261 (2 mg/kg) via intraperitoneal	The treatment with caffeine led to an improvement in balance disorders in mice treated with 6-OHDA. These results could be obtained through the inhibition of presynaptic A_2A_R, as demonstrated by the use of its antagonist, SCH 58261.	[103]
Long Evans rats	6-OHDA (12 μg) unilaterally via intraperitoneal	Caffeine, SCH 5826, L-DOPA, N^6^-Cyclopentyladenosine and 8-Cyclopentyltheophylline	Caffeine (15 mg/kg), SCH 58261 (2 mg/kg), L- DOPA (8 mg/kg) N^6^-Cyclopentyladenosine (0.03–0.2 mg/kg) and 8-Cyclopentyltheophylline (3–7 mg/kg) via systemic intraperitoneal	The caffeine treatment associated with L-DOPA in rats induced with 6-OHDA improved the motor activity. These improvements could be obtained through the inhibition of A_2A_R, as demonstrated by the use of its antagonist SCH 5826.	[104]
Sprague Dawley rats	6-OHDA (2 μg/μL) unilateral infusion into the nigrostriatal	L-DOPA, Caffeine, SCH 412348, Istradefillin and Vipadenant	L-DOPA (6 mg/kg), Caffeine (30 mg/kg), SCH 412348 (3 mg/kg), Istradefillin (3 mg/kg) and Vipadenant (10 mg/kg) after 14 days from the injury with L-DOPA and A_2A_R antagonists for 19–22 days	Chronic treatment with caffeine or A_2A_R antagonists (SCH 412348, vipadenant, or istradefillin) does not induce dyskinetic activity in rats treated with 6-OHDA, unlike L-DOPA.	[105]
C57BL/6 (*A_1_*^−/−^, *A_2A_*^+/+^) KO, (*A_1_*^+/+^, *A_2A_*^−/−^) KO and (*A_1_*^−/−^, *A_2A_*^−/−^) KO mice	6-OHDA (10 μg) stereotactic unilateral injection	Caffeine and L-DOPA	Caffeine (3–15 mg/kg) and L-DOPA (2 mg/kg) via intraperitoneal with L-DOPA for 2–3 weeks after 14 days from the injury and 10 min before the administration of L-DOPA with two doses intraperitoneal of caffeine	Caffeine did not alleviate L-DOPA-induced dyskinesia, probably due to its general motor stimulation actions. The simultaneous blocking of A_1_R and A_2A_R, like caffeine, did not improve dyskinesia compared to the use of a specific A_1_R or A_2A_R antagonist alone.	[106]
Wistar rats	ROT (1.5 mg/kg) via intraperitoneal for 45 days	Caffeine	30 mg/kg before or after the induction of ROT	Caffeine treatment restored dopamine levels in the corpus striatum and prevented motor and muscle deficits induced by ROT. Caffeine reduced the level of MDA and oxidative stress.	[107]
C57BL/6NCrl mice	PQ (10 mg/kg) and MB (30 mg/kg) via intraperitoneal 10 min after caffeine treatment	Caffeine	5 or 20 mg/kg via intraperitoneal	Caffeine treatment prevented the neurodegeneration of dopaminergic neurons induced by chronic pesticide exposure.	[108]
Wistar rats	Reserpine (5 mg/kg) via intraperitoneal	Caffeine and THP	Caffeine (1 mg/kg) and THP (0.1 mg/kg), 24 h after the injury, the animals were treated alone with caffeine or THP, or caffeine and THP combined	In reserpine-induced rats, the co-treatment of caffeine and THP led to the recovery of motor and exploratory activities. The treatments with caffeine or THP did not invert hypokinesia induced by reserpine.	[111]

MPTP: N-methyl-4-phenyl-1,2,3,6-tetrahydropyridine; A_2A_R: adenosine _2A_ receptor; DMPX: 3,7-dimethyl-1-propargylxanthine; KW-6002: istradefylline; BBB: blood brain barrier; PD: Parkinson’s Disease; 6-OHDA: 6-hydroxydopamine; DOPAC: 3,4-dihydroxyphenylacetic acid; CSC: 8-(-3-chlorostyryl)-caffeine (CSC); L-DOPA: L-3,4-dihydroxyphenylalanine; AIMs: abnormal involuntary movements; KO: knockout; *A_1_*^−/−^, *A_2A_*^+/+^: A_1_R KO; *A_1_*^+/+^, *A_2A_*^−/−^: A_2A_R KO; *A_1_*^−/−^, *A_2A_*^−/−^: A_1_R-A_2A_R KO; ARs: adenosine receptors; A_1_R: adenosine _1_ receptor; ROT: rotenone; MDA: malondialdehyde; SOD: superoxide dismutase; PQ: paraquat; MB: maneb; GSH-S-transferase: glutathione-S-transferase; THP: trihexyphenidyl.

**Table 2:** Summary of the clinical studies that show the neuroprotective effects of caffeine in PD patients and prospective studies that highlight the direct correlation between the consumption of caffeine and the low risk of PD.

Title Study	Type Study	Patients	Age	Outcome	Results	Ref.
Caffeine for Motor Manifestations of Parkinson’s Disease (NCT01190735)	Complete phase 2 clinical study	28	aged 18 years and older	The aim of the study was to evaluate the tolerability and efficacy of the treatment at increasing doses of caffeine (100–200–300–500 mg).	NA	-
Caffeine for Excessive Daytime Somnolence in Parkinson’s Disease (NCT00459420)	Double-blind controlled phase 2/3 complete study	58	aged 18 years and older	The study evaluated the effects of caffeine in idiopathic PD patients treated daily with caffeine (100 mg) and for the following 3 weeks with caffeine (200 mg).	Caffeine treatment led to a reduction in the ESS score and the improvement of the Clinical Global Impression of Change outcomes and UPDRS.	[112]
Caffeine as a Therapy for Parkinson’s Disease (NCT01738178)	Complete phase 3 clinical study	121	aged 45–70 years	The aim of the study was to evaluate the effects of caffeine (200 mg) in idiopathic PD patients.	Caffeine did not improve motor parkinsonism, while it led to a slight improvement in sleepiness. Patients taking for long-time caffeine had a slight increase in dyskinesia and a worsening in cognitive tests.	[113]
Prospective study of caffeine consumption and risk of Parkinson’s disease in men and women	Prospective cohort study	10 men and 16 women	aged 40–75 years and aged 30–55 years	The study assessed the relationship between caffeine consumption with PD risk.	Moderate coffee consumption reduced the PD risk more in men than women.	[17]
Coffee Consumption, Gender, and Parkinson’s Disease Mortality in the Cancer Prevention Study II Cohort: The Modifying Effects of Estrogen	Cohort study	909 men and 340 women	average aged 57 years men and aged 56 years women	The aim of the study was to evaluate the relationship between caffeine consumption and mortality rate for PD.	Caffeine consumption associated with smoking and alcohol led to a decrease in PD risk and a reduction in the rate of mortality.	[114]
Caffeine and Risk of Parkinson’s Disease in a Large Cohort of Men and Women	Prospective study	197 men and 120 women	mean age 75 years men and 74 women	The study evaluated the relationship between caffeine consumption and PD risk.	Caffeine consumption reduced the risk of PD onset in both sexes.	[115]
Associations of Lower Caffeine Intake and Plasma Urate Levels with Idiopathic Parkinson’s Disease in the Harvard Biomarkers Study	Case-control study	566	average age 67 years	The aim of the study was to assess the effects of caffeine consumption and PD risk.	Caffeine consumption was associated with a lower risk of onset of idiopathic PD in the sex-independent way.	[116]
Association of Coffee and Caffeine Intake with the Risk of Parkinson Disease	Cohort study	8004	aged 45–68 years	The study assessed the relationship between caffeine consumption and PD risk.	The habitual caffeine consumers showed PD risk to be 5 times lower compared to non-coffee consumers.	[15]
Risk Factors for Parkinson’s Disease: The Leisure World Cohort Study	Case-control study	395	mean ages 75 years	The aim of the study was to evaluate the effects on caffeine consumption, as well as smoking and other risk factors related to PD risk.	Coffee consumption and smoking led to a reduction in the PD risk.	[117]
Coffee and tea consumption and the risk of Parkinson’s disease	Cohort study	200	aged 25–64 years	The study assessed the relationship between caffeine consumption and PD risk.	The higher caffeine consumption reduces the PD risk, especially in the 25–49 age range.	[118]
Prospective Study of Coffee Consumption and Risk of Parkinson’s Disease	Prospective Study	101	aged 50–79 years	The aim of the study was to analyze coffee consumption on the incidence of PD.	The daily consumers of coffee showed a lower risk of PD onset compared to non-coffee drinkers.	[16]
Differential Effects of Black Versus Green Tea on Risk of Parkinson’s Disease in the Singapore Chinese Health Study	Cohort study	157	aged 45–74 years	The study evaluated the relationship between caffeine consumption and PD risk.	The habitual coffee consumers or black tea, but not of green tea showed a low risk of developing PD.	[119]
A meta-analysis of coffee drinking, cigarette smoking, and the risk of Parkinson’s disease	Meta-analysis study	18,605	NA	The aim of the study was to assess coffee consumption and PD risk.	The daily coffee consumers showed a low PD risk compared to non-consumers.	[120]
Dietary and Lifestyle Variables in Relation to Incidence of Parkinson’s Disease in Greece	Cohort study	118	aged 20–86 years	The aim of the study was to examine the relationship between caffeine and eating habits as well as lifestyle and PD risk.	Caffeine consumption was associated with a lower risk of PD.	[121]
Caffeine Intake, Smoking, and Risk of Parkinson Disease in Men and Women	Cohort study	318,260	aged 50–71 years	The study was to evaluate the relationship between caffeine intake and PD risk.	Caffeine intake reduced the risk of PD onset independently by gender.	[122]

NA: not available; ESS: Epworth Sleepiness Scale; UPDRS: Unified Parkinson’s Disease Rating Scale; HC: healthy controls; PD: Parkinson’s Disease.

## Abbreviations

PD	Parkinson’s Disease
α-Syn	α-synuclein
ROT	rotenone
PQ	paraquat
MB	maneb
ROS	reactive oxygen species
H_2_O_2_	hydrogen peroxide
GSH	Glutathione
BBB	blood brain barrier
CNS	central nervous system
ARs	adenosine receptors
MAO-B	monoamine oxidase-B
Nrf2	nuclear factor erythroid 2-related factor 2
PGC-1α	peroxisome proliferator-activated receptor-gamma coactivator 1-α
KO	knockout
PDE	phosphodiesterase
A_2A_R	adenosine _2A_ receptor
PKA	protein kinase A
TNF-α	tumor necrosis factor alpha
L-DOPA	L-3,4-dihydroxyphenylalanine
GABA	γ-aminobutyric acid
A_1_R	adenosine _1_ receptor
MPTP	N-methyl-4-phenyl-1,2,3,6-tetrahydropyridine
6-OHDA	6-hydroxydopamine
qRT-PCR	quantitative real-time polymerase chain reaction
DOPAC	3,4 dihydroxyphenylacetic acid
CSC	8-(-3-chlorostyryl)-caffeine
AIMs	abnormal involuntary movements
THP	trihexyphenidyl
ESS	Epworth Sleepiness Scale
UPDRS	unified Parkinson’s Disease rating scale
HC	healthy control
HPFS	Study of Health Professionals
NHS	Nurses’ Health Study
